# Full-endoscopic foraminoplasty for highly down-migrated lumbar disc herniation

**DOI:** 10.1186/s12891-022-05254-4

**Published:** 2022-03-29

**Authors:** Hanhua Cai, Chunhua Liu, Haibin Lin, Zhiqiang Wu, Xuanhuang Chen, Huaizhi Zhang

**Affiliations:** 1 Department of Orthopaedic Surgery, Affiliated Hospital of Putian University, Putian, Fujian Province China; 2 Department of Spinal Surgery, Quanzhou Orthopedic-Traumatological Hospital, Fujian University of Traditional Chinese Medicine, Quanzhou, Fujian Province China

**Keywords:** Full-endoscopic discectomy, Foraminoplasty, Downward migration, Lumbar disc herniation

## Abstract

**Background and study aims:**

Multiple surgical approaches have been studied and accepted for the removal of highly downward migrated lumbar disc herniation (LDH). Here, we investigated the efficacy and safety of full-endoscopic foraminoplasty for highly downward migrated LDH.

**Patients and methods:**

Thirty-seven patients with highly down-migrated LDH treated by the full-endoscopic foraminoplasty between January 2018 and January 2020 were retrospectively investigated. Clinical parameters were evaluated preoperatively and 1, 6, and 12 months postoperatively, using pre- and post-operative Oswestry Disability Index (ODI) scores for functional improvement, visual analog scale (VAS) for leg and back pain, and modified MacNab criteria for patients satisfactory.

**Results:**

Thirty-seven patients with highly downward migrated LDH were successfully removed via the transforaminal full-endoscopic discectomy. The average VAS back and leg pain scores were significantly reduced from 7.41 ± 1.17 and 8.68 ± 1.06 before operation to 3.14 ± 0.89 and 2.70 ± 0.46 at postoperative 1 month, and 1.76 ± 0.59 and 0.92 ± 0.28 at postoperative 12 months, respectively (*P* < 0.05). The average ODI scores were reduced from 92.86 ± 6.41 to 15.30 ± 4.43 at postoperative 1 month, and 9.81 ± 3.24 at postoperative 12 months (*P* < 0.05). Based on the modifed MacNab criteria, 36 out of 37 patients (97.30%) were rated as excellent or good outcomes.

**Conclusion:**

The full-endoscopic foraminoplasty can be used successfully for surgical removal of high grade down-migrated LDH, and it could serve as an efficient alternative technique for patients with highly downward migrated LDH.

**Supplementary Information:**

The online version contains supplementary material available at 10.1186/s12891-022-05254-4.

## Introduction

Lumbar disc herniation (LDH), extending beyond the inferior or superior margin of the intervertebral disc in downward or upward direction, was generally described as disc migration [1, 2]. According to the Lee’s classification depending on the distance from the disc space, the extent of down-migrated disc was classified into two zones: near-downward and far-downward [1]. Typically, patients with the compression of the dural sac or nerve roots will progress to the potential neurological symptoms, which adversely affect the quality of patients’ daily life [3, 4].

Several endoscopic discectomy techniques that achieve satisfying clinical results with less trauma have been extensively researched and used for LDH [4–7]. Despite the popularity of endoscopic discectomy techniques, the value of the full-endoscopic foraminoplasty for surgical removal of highly downward migrated LDH has yet to be well-demonstrated.

As the concept of endoscopic technique has been shifted from intra-discal decompression to adjustable alternative decompression, full-endoscopic discectomy for LDH has been developed and extensively used [6, 8, 9]. We therefore investigated the efficacy and safety of full-endoscopic foraminoplasty for highly downward migrated LDH.

## Patients and methods

Ethics committee approval and informed consent for each patient were obtained in this series.

### Patient population

**Fig. 1:**
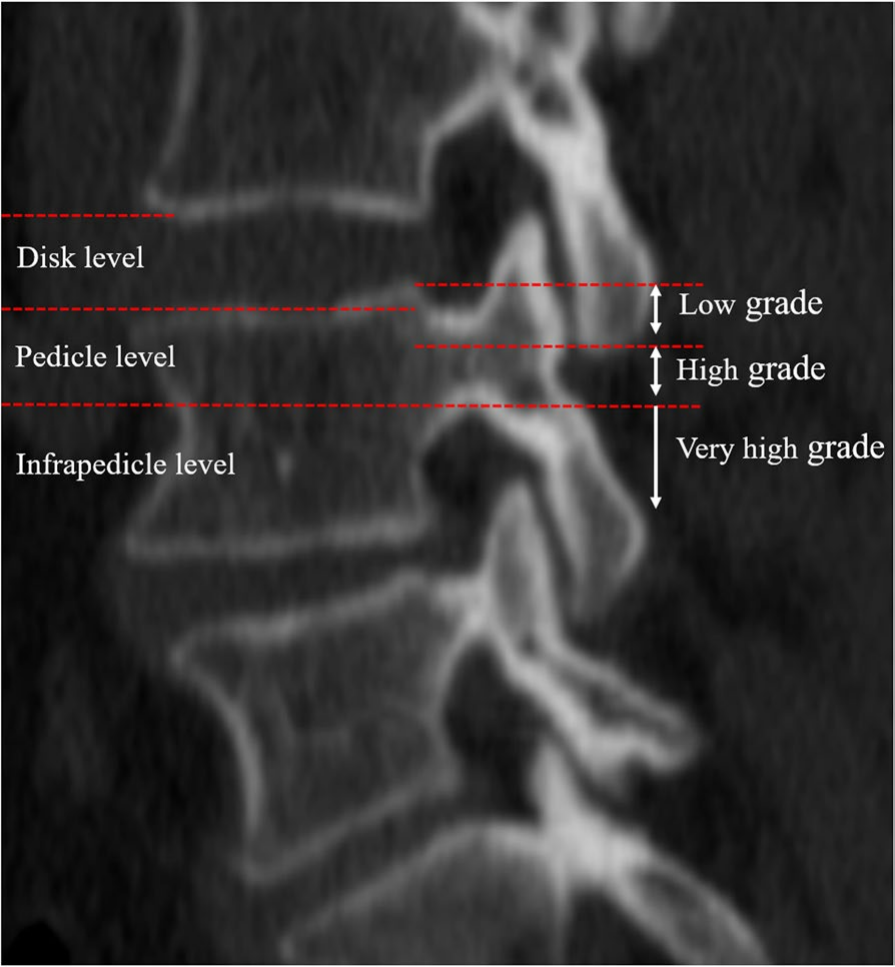
The extent of down-migrated disc: Zone 1, low-grade downward: herniations within the height of the posterior margin of the disc in downward direction; Zone 2, high-grade downward: herniations extending beyond the height of the posterior margin of the disc in downward direction; Zone 3, very high-grade downward: herniations extending beyond the inferior margin of lower pedicle

Between January 2018 and January 2020, 37 consecutive cases with highly downward migrated LDH were retrospectively investigated and included in the present study. Of them, herniations extending beyond the height of the posterior margin of the disc are defined as high-grade downward migrations [1, 10]. Herniations extending beyond the inferior margin of the pedicle are defined as very high-grade downward migrations [5]. The full-endoscopic foraminoplasty under local anesthesia was conducted for surgical removal of highly down-migrated LDH by the experienced spine surgeon. The schematic representation of the levels of herniation and 3 zones was presented in the Fig. 1.

The eligibility criteria were as follows: radiating pain and numbness in the unilateral lower extremity; single-level highly down-migrated LDH verified by imageology examination; patients’ clinical symptoms in accordance with imageology detection; ineffectual conservative treatment for at least 12 weeks, except for the patient was in severe pain that affected their daily life; and no surgical history at the same level. The exclusion criteria included non-downward migrated LDH, lumbar spinal stenosis, multiple level LDH, lumbar instability, and lumbar spinal infection or tumor.

### Surgical technique

**Fig. 2:**
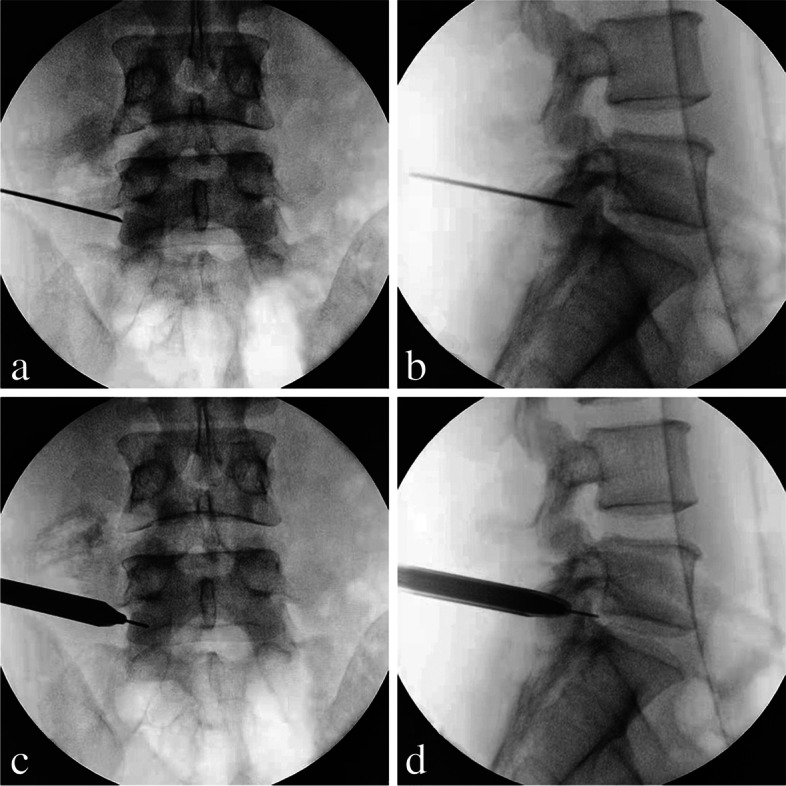
**a** Anteroposterior (AP) fluoroscopic view showing the needle tip located at the exterior margin of the superior articular process. **b** Lateral fluoroscopic view showing the needle tip located at the tip of the superior articular process. **c** AP view showing the working cannula located at the exterior margin of the superior articular process. **d** Lateral fluoroscopic view showing the working cannula located at the tip of the superior articular process

The patients were adjusted to prone position with a soft cushion under their abdomen. Based on the C-arm fluoroscopy, the body surface projection line of the target segment and the spinous process midline, as well as the puncture route were marked on the patient’s back. The surgical procedure under local anesthesia was performed for all patients. The puncture route on the lateral fluoroscopic view was set from the apex of the upper articular process to the posterior upper margin of the inferior vertebral body. Then, an 18-gauge needle was slowly advanced to the superolateral region of the superior articular process (Fig. 2a-b). The skin entry point was slightly located at upper margin of the disc level, making an angle of approximately 10–30° with the superior articular process. During the puncture pathway, the skin entry point, subcutaneous tissue, and peripheral facet joint were infiltrated for anesthesia with the mixture of lidocaine and ropivacaine. After the precise location of the needle was confirmed by C-arm fluoroscopy, the needle core was pulled out and the guide wire was inserted.

**Fig. 3:**
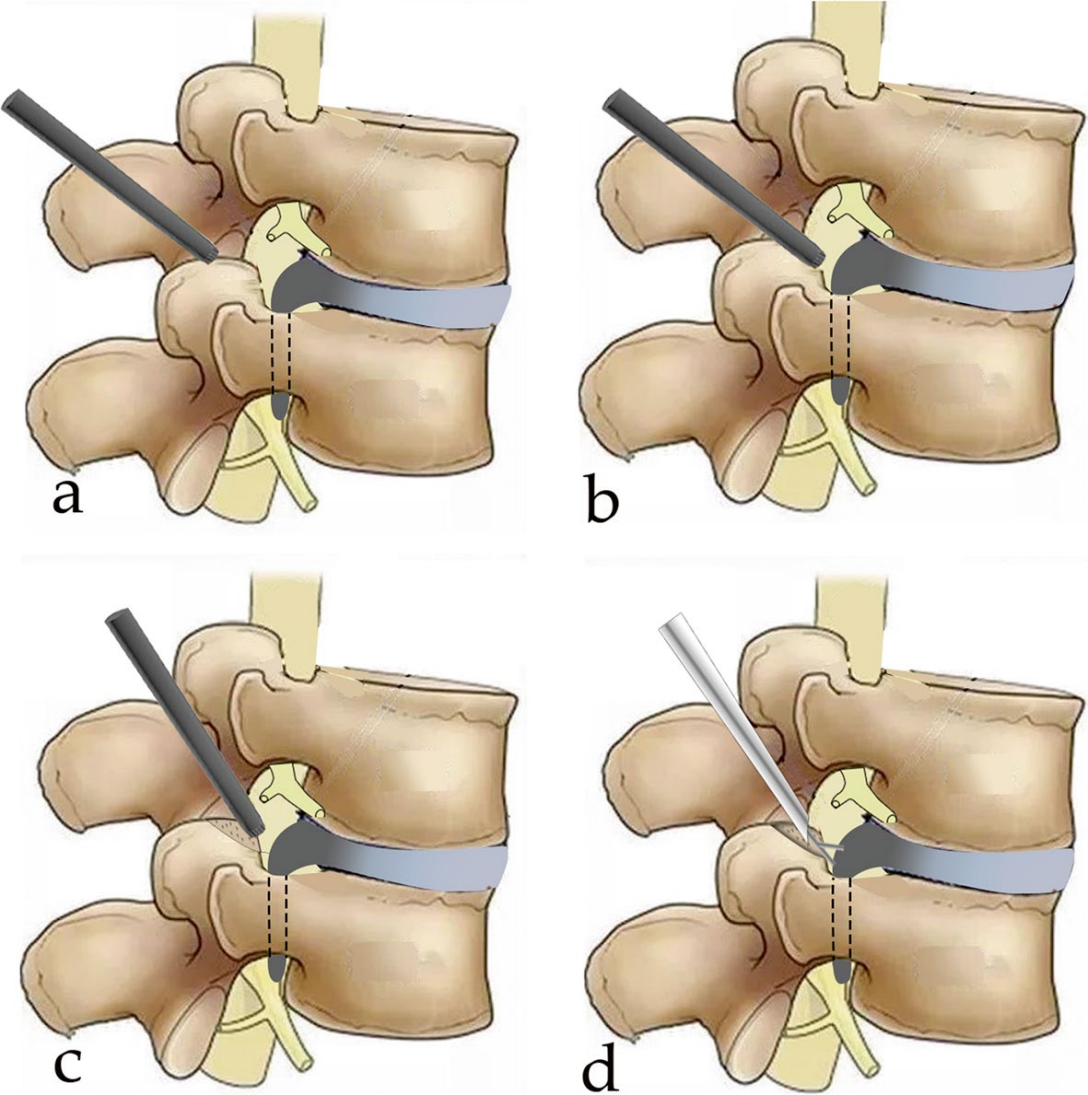
Surgical procedures of highly downward-migrated disc removal. **a** Placement of the endoscopic trephine; **b** The ventral superior articular process was partially resected using the endoscopic trephine; **c** The working cannula was directed downward to create an angle of a cranial-to-caudal approach; **d** The endoscopic forceps were scrupulously used to remove high grade down-migrated mass

Subsequently, an about 7 mm incision, approximately 8–12 cm off the midline on the line of the puncture route, was made. Following a staged dilation along the guide wire, the working cannula (8.4 mm) (Fig. 2c-d) and endoscopic trephine (7.5 mm) (Fig. 3a) (Guanlong, Shandong, China) was placed, which were located at the exterior margin of the superior articular process from anteroposterior view and at the tip of the superior articular process from lateral fluoroscopic view. Then, the 30-degree endoscope (6.3 mm) (Joimax, Karlsruhe, Germany) was introduced and inserted into the working cannula. Under endoscopic direct vision, the soft tissue around the peripheral facet joint was ablated by the bipolar radiofrequency electrode (Guanlong, Shandong, China), and the ventral superior articular process was partially resected to enlarge the intervertebral foramen, using the endoscopic trephine (Fig. 3b). The working cannula and endoscope were further pushed toward the lumbar canal, and the ligamentum flavum was directly identified and then removed with the use of the endoscopic forceps. At this point, the traversing nerve root, squeezed by the annulus fibrosus, was observed. The dense adhesions between fibrous anchoring tissues and nerve root were released with bipolar radiofrequency electrode.

In order to provide a sufficient decompression of the traversing nerve root, full-endoscopic discectomy under continuous visualization was preliminarily performed. Once the release of intra-discal and epidural space is sufficient, the highly down-migrated fragments can be detected clearly, and the epidural operating space can be achieved for endoscopic instruments. Subsequently, the working cannula was directed downward to create an angle of a cranial-to-caudal approach (Fig. 3c). Thereafter, endoscopic forceps were scrupulously used to remove high grade down-migrated mass (Fig. 3d). Following a careful endoscopic exploration, thorough decompression was confirmed when the traversing nerve root was repeatedly seen moving freely via the Valsalva maneuver. After active bleeding was controlled with the use of bipolar radiofrequency electrode, the endoscope with the working cannula was dislodged, and a piece of sterile dressing was placed on the stitched incision.

### Clinical evaluation

Data for baseline characteristics and perioperative variables were recorded. Leg and back pain were evaluated using visual analog scale (VAS) [11] (range, 0–10) and neurologic function were evaluated using Oswestry disability index score (ODI) [12] (range, 0–100). Patient’s satisfaction was concurrently assessed on the basis of the modified MacNab criteria [7, 13]. Clinical parameters were scheduled preoperatively and 1, 6, and 12 months postoperatively.

### Statistical analysis

Enumeration and quantitative data, respectively, were descriptively presented as percentage and mean (standard deviation). The clinical outcomes, based on the modified MacNab criteria, were categorized as excellent, good, fair, and poor. The difference in terms of pre- and post-operative VAS or ODI scores at different time points was calculated statistically by repeated measures analysis of variance. Statistical analyses for collected data were calculated with the assistance of IBM SPSS Statistics 20. *P* value less than 0.05 indicates a statistical difference.

## Results

### Base demographic and clinical characteristics

21 cases with highly down-migrated and 16 cases with very highly down-migrated were included. This series included three cases located at L2/3, three cases at L3/4, fifteen cases at L4/5, and sixteen cases at L5/S1. There were 12 females and 25 males. The age ranged from 30 to 63 years, with a mean of 46.43 ± 9.15 years. During surgery, the blood loss was not measured in these patients. The average fluoroscopy time and operation time were 6.03 ± 1.91 times and 92.70 ± 24.07 min, respectively. The mean hospitalization time was 6.27 ± 2.07 days. The patient-related information was recorded and summarized in Table 1.

**Table 1:** Base demographic and clinical characteristics

Variables	Outcomes
Gender (male: female) (n)	25:12
Age (mean ± SD) (years)	46.43 ± 9.15
Herniated segment (n)	
L2/3	3
L3/4	3
L4/5	15
L5/S1	16
Migration extent	
Highly	21
Very highly	16
Operation time (mean ± SD) (minutes)	92.70 ± 24.07
Fluoroscopy times (mean ± SD) (times)	6.03 ± 1.91
Hospital stay (mean ± SD) (days)	6.27 ± 2.07
Complications (n)	
Recurrence	1
Postoperative dysesthesia	4

*SD* Standard deviation

### Clinical outcomes

#### Pain measurement

Detailed results of clinical outcomes were present in Table 2. Thirty-seven patients with highly downward migrated LDH were successfully removed. All patients’ back and leg pain was relieved after operation. The average VAS back pain scores had reduced significantly, from 7.41 ± 1.17 before operation to 3.14 ± 0.89 at postoperative 1 month, 2.59 ± 0.69 at postoperative 6 months, and 1.76 ± 0.59 at postoperative 12 months (*P* < 0.05), as shown in supplementary Fig. 1. Similarly, the average VAS leg pain scores had also reduced significantly, from 8.68 ± 1.06 before operation to 2.70 ± 0.46 at postoperative 1 month, 1.81 ± 0.46 at postoperative 6 months, and 0.92 ± 0.28 at postoperative 12 months (*P* < 0.05), as shown in Supplementary Fig. 2.

**Table 2:** Detailed results of clinical outcomes

Variables	Pre-op	1 m post-op	6 m post-op	12 m post-op	Statistic F	p
VAS back pain	7.41 ± 1.17	3.14 ± 0.89	2.59 ± 0.69	1.76 ± 0.59	351.94	0.00
VAS leg pain	8.68 ± 1.06	2.70 ± 0.46	1.81 ± 0.46	0.92 ± 0.28	1147.39	0.00
ODI scores	92.86 ± 6.41	15.30 ± 4.43	12.03 ± 3.75	9.81 ± 3.24	5987.71	0.00

*VAS* Visual analog scale, *ODI* Oswestry disability index

#### Assessment of disability

As for neurological function, the average ODI scores had reduced significantly, from 92.86 ± 6.41 before operation to 15.30 ± 4.43 at postoperative 1 month, 12.03 ± 3.75 at postoperative 6 months, and 9.81 ± 3.24 at postoperative 12 months (*P* < 0.05), as shown in supplementary Fig. 3. Based on the modifed MacNab criteria, 36 out of 37 patients (97.30%) were rated as excellent or good outcomes, as shown in Table 3.

**Table 3:** Modifed MacNab criteria

	Excellent	Good	Fair	Poor
Patients (n)	21	15	0	1
Percentage (%)	56.76	40.54	0	2.70

#### Complications

Lumbar radicular pain at the same segment was observed in one patient at 3 days postoperatively, which was successfully relieved via a second transforaminal full-endoscopic discectomy. Additionally, four patients had early postoperative dysesthesia of the traversing root that was satisfactorily resolved with neurotrophic drugs for 2 weeks. No occurrence of nerve root injury or dura tear was observed.

#### Illustrative case

**Fig. 4:**
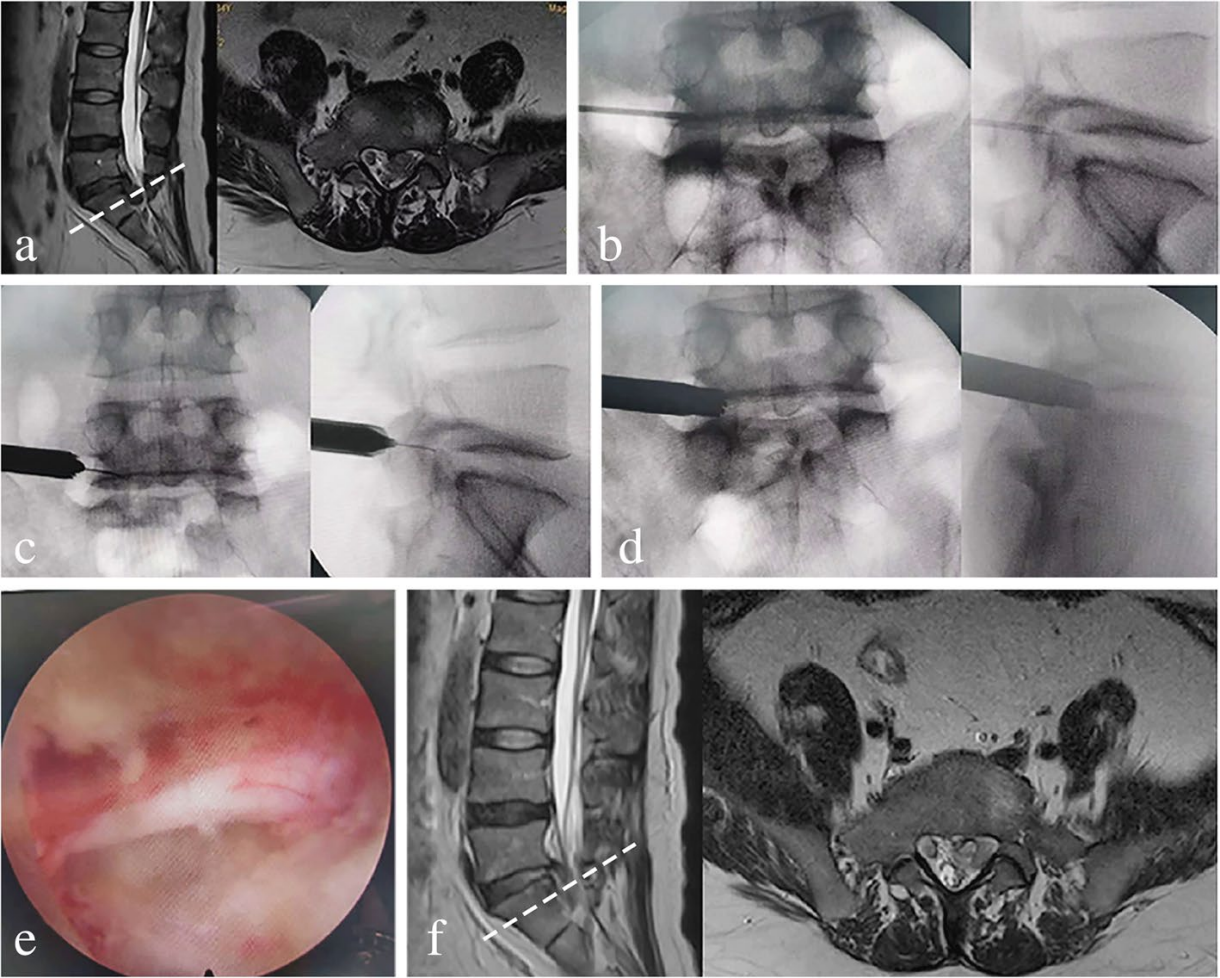
Thirty-four-year-old man with highly downward-migrated LDH, whose complaint was low back and left lower limb pain. The full-endoscopic foraminoplasty was performed. **a** Preoperative sagittal and axial T2 MRI showing a highly down-migrated disc from L5 to S1. **b** The needle tip located at the exterior margin and tip of the superior articular process. **c** The working cannula located at the exterior margin and tip of the superior articular process. **d** The ventral superior articular process was partially resected using the endoscopic trephine. **e** Thorough decompression was obtained. **f** Postoperative sagittal and axial T2 MRI showing successful surgical removal of the downward migrated fragments

**Fig. 5:**
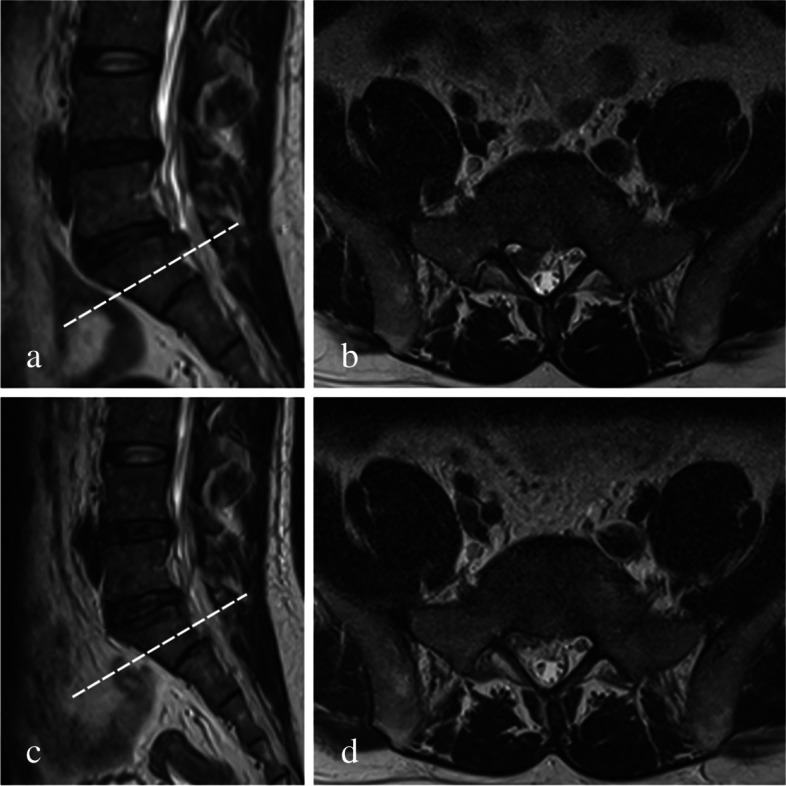
**a** Preoperative sagittal and (**b**) axial T2 MRI showing a highly down-migrated disc from L5 to S1. **c** Postoperative sagittal and (**d**) axial T2 MRI showing successful surgical removal of the downward migrated fragments

**Fig. 6:**
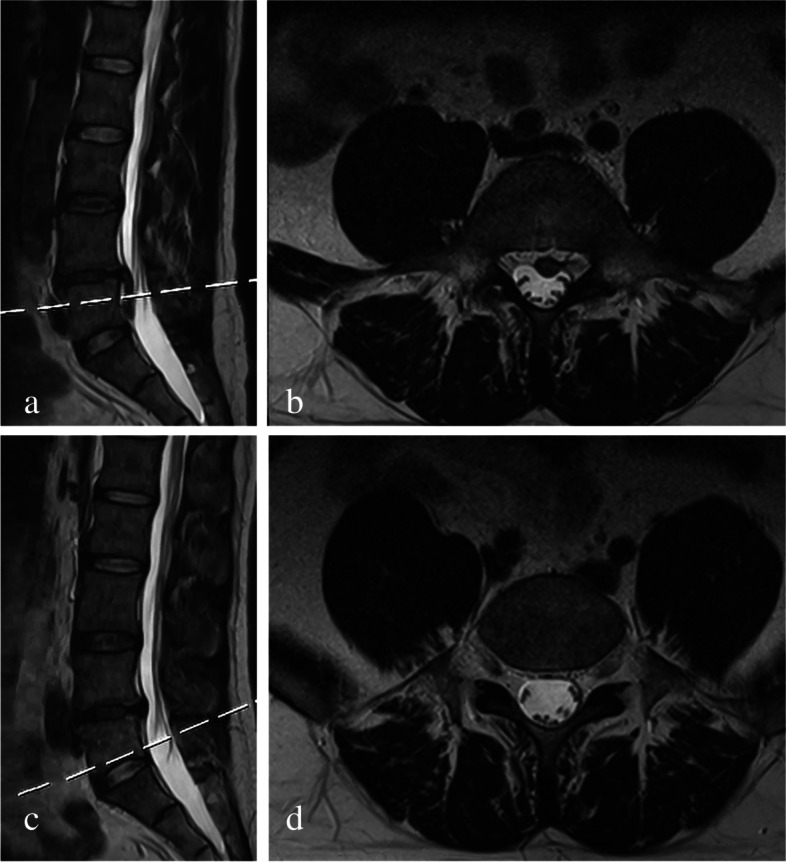
**a** Preoperative sagittal and (**b**) axial T2 MRI showing a very highly down-migrated disc from L4 to L5. **c** Postoperative sagittal and (**d**) axial T2 MRI showing successful surgical removal of the downward migrated fragments

**Fig. 7:**
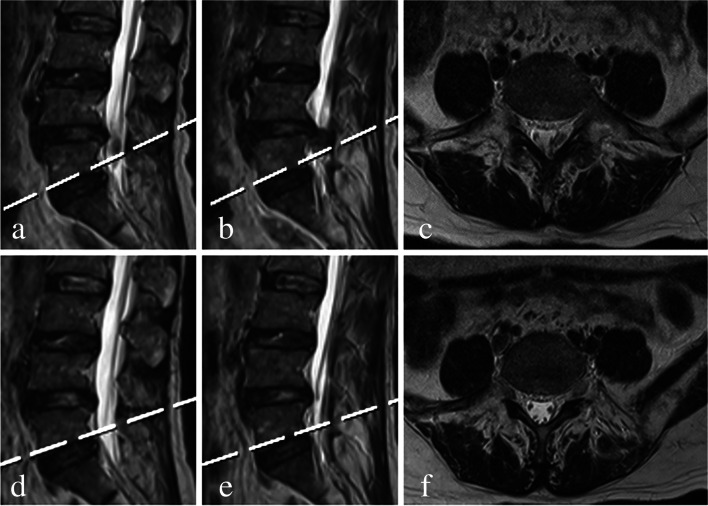
**a**, **b** Preoperative sagittal and (**c**) axial T2 MRI showing a very highly down-migrated disc from L4 to L5. **d**, **e** Postoperative sagittal and (**f**) axial T2 MRI showing successful surgical removal of the downward migrated fragments

Figure 4 presented the preoperative, postoperative images and intraoperative fluoroscopic views of a thirty-four-year-old man who was treated with full-endoscopic foraminoplasty. An illustrative case of the highly down-migrated disc was presented in Fig. 5a-d, and the very highly down-migrated disc in Figs. 6a-d and 7a-f.

## Discussion

Downward migrated LDH is sometimes discontinuous and sequestrated from the origin intervertebral disc, and it is generally fragmented into small pieces [2, 5, 14]. The posterior longitudinal ligament can be breached by the disc migrated sequestrations, which can drift cranially or caudally into the anterior epidural space, with an estimated prevalence of 35 to 70% [15, 16]. Conventionally, the removal of these highly down-migrated sequestrations via the traditional surgical approaches requires extensive soft tissue dissection, even at the cost of sacrificing part of the lamina and facet joints, which may result in potential lumbar instability and associated complications [17, 18]. Thus, in order to minimize the possibility of iatrogenic lumbar instability, an experienced surgeon may create a bony opening through the lamina [18, 19]. Nevertheless, it is difficult to reach and remove thoroughly these migrated fragments, since some sequestrations around the corner cannot completely be examined.

Specifically, several surgical approaches have been developed to easily get through the sequestrated LDH. In 2008, Choi et al. [20] conducted a retrospective analysis of 59 patients with highly migrated intracanal disc herniations who underwent percutaneous endoscopic approach using foraminoplastic technique, and found that the outcomes of foraminoplastic-PELD were comparable to those of conventional open procedures. Also, the author described patients with highly downward migrated LDH had superior outcomes compared with cases with highly downward migrated LDH. Kim et al. [5] in 2012 investigated surgical outcomes of interlaminar PELD for highly migrated LDH in 18 patients. The disc sequestrations were migrated inferiorly in 11 cases and superiorly in 7 cases. Of them, 12 patients had excellent outcomes in 66.67% of cases, good in 3 patients. But in one case, dural tear without cerebrospinal fluid leakage was suspected. Lin et al. [21] described their experience with accessing a highly migrated LDH using full endoscopic technique via a translaminar keyhole approach in 13 patients. Although these patients presented with highly upward disc migration and only two cases located at L5/S1, the satisfactory outcomes were observed in 92.3% of cases, which was consistent with our results. The difference was that the patients in our series were with a younger average age.

Nonetheless, the full-endoscopic foraminoplasty has not yet been well clarified as an effective removal for high-grade downward migrated LDH. Our experience in the present series demonstrates an effective and safe procedure for achieving majority removal of a highly downward migrated LDH in our patients. The clinical findings reveal that the effectiveness with respect to pain subsides, perioperative parameters, and neurological functional recovery was similar to those achieved with aforementioned approaches, which efficiently avoid an open limited incision, significant soft tissue dissection, and irreversible bony destruction. In terms of safety, the entire surgical procedure was performed under direct vision, which has the advantages of smaller wounds and less bleeding, and has the potential to decrease the likelihood of nerve damage and maximize rehabilitation potential with faster resumption of activities while minimizing hospitalization time. Ruetten et al. analyzed a series of cases who underwent interlaminar PELD, and found that 2% of the patients had nerve damage [22]. Fortunately, the retraction injury that can happen in posterior surgery was not observed in our series.

Technically, the full-endoscopic foraminoplasty can directly access initial site of a herniated disc and reach highly down-migrated fragments through adjusting the working cannula, possibly indicating it can be used as an alternative to traditional open discectomy since it is not restricted by the high iliac crest. Moreover, the area of operative exploration is very bright illumination and fully visualized, since the endoscope provides clearly high-resolution image under direct vision. It creates favorable conditions to avoid nerve damage and remove the migrated disc as completely as possible. Importantly, the radiation exposure and fluoroscopy time were significantly reduced because the establishment of working cannula was completed under continuous visualization, as well as foraminoplasty.

Importantly, several negative aspects of the full-endoscopic foraminoplasty should also be emphasized. First, complete removal of the migrated sequestrations that were separated into multiple pieces may not be attainable [2, 5]. However, the existence of the hidden fragment can be acceptable if there were no clinical symptoms after operation. With the time extension, some residual disc material can even be resorbed [5, 20]. Secondly, despite the skillful full-endoscopic transforaminal technique, traction or exfoliation between the nerve root and the migrated disc may cause additional dural sac or nerve root damage, especially in case with tight adhesion. Therefore, careful attentions should be taken to protect the dural sac or nerve root during the operation. Thirdly, partial surgical resection of the ventral superior articular process is a time-consuming procedure when foraminoplasty using the endoscopic trephine, and it could result in bleeding on the bone surface, which is hardly controlled with an aid of bipolar radiofrequency coagulators [23, 24]. Additionally, a large proportion of lumbar spine diseases may not be suitable for this technique, such as vertebral endplate (Modic) change, metastatic vertebral lesions, and central lumbar spinal stenosis.

Several shortcomings of the present study should be mentioned. First, as this is non randomized and not matched to control/cohort study, solid conclusion may not be drawn about the efficacy and advantages of the technique. Secondly, although all included patients’ radiating pain were relieved immediately after operation, a minority of patients were not reviewed by MRI during the follow up. There may be the existence of the hidden fragment. Thirdly, a limited number of cases with short-term follow-up may decrease the credibility of the findings that drawn from this series. Therefore, we believe that a larger number of cases is necessary to investigate the long-term efficacy of transforaminal full-endoscopic discectomy for highly down-migrated LDH.

## Conclusion

Although surgical removal of highly downward migrated LDH is challenging, our favorable clinical results favor the full-endoscopic foraminoplasty performed in this series. We thus conclude that transforaminal full-endoscopic discectomy is an efficient alternative technique for surgical removal of highly downward migrated LDH. Nonetheless, further large sample size, multicenter comparative studies with long-term follow-up period need to validate our encouraging outcomes.

## Supplementary Information

**Additional file 1.**

## Data Availability

The datasets presented in the current study are not publicly available since limitations of ethical approval involving patients’ data but are available from the corresponding author or co-author on reasonable request.

## Abbreviations

LDH: Lumbar disc herniation
ODI: Oswestry Disability Index
VAS: Visual analogue scale
MRI: Magnetic resonance imaging
AP: Anteroposterior
SD: Standard deviation
