# Supplementary material for: Full-endoscopic foraminoplasty for highly down-migrated lumbar disc herniation
Source: BMC Musculoskelet Disord. 2022 Mar 29;23:303. doi: 10.1186/s12891-022-05254-4 (PMC8966215; doi:10.1186/s12891-022-05254-4)
Supplement: Supplementary file 1 — Additional file 1. [file 12891_2022_5254_MOESM1_ESM.docx]

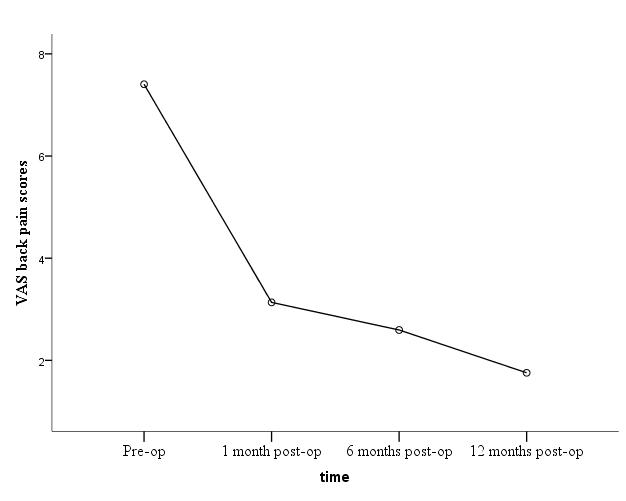


**Supplementary Fig 1**. Visual analog scale (VAS) for back pain scores over time.


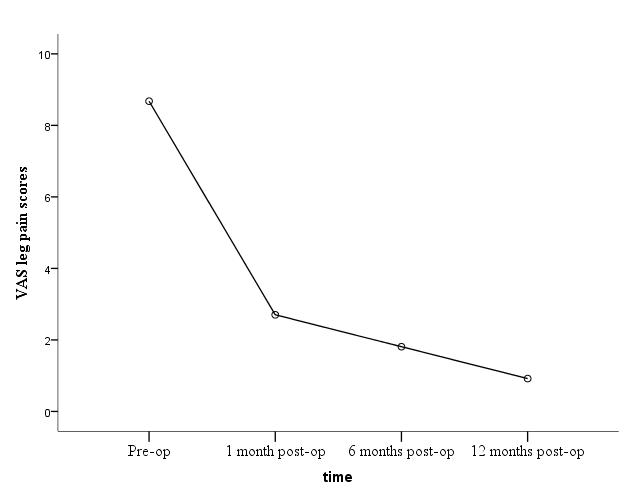


**Supplementary Fig 2**. Visual analog scale (VAS) for leg pain scores over time.


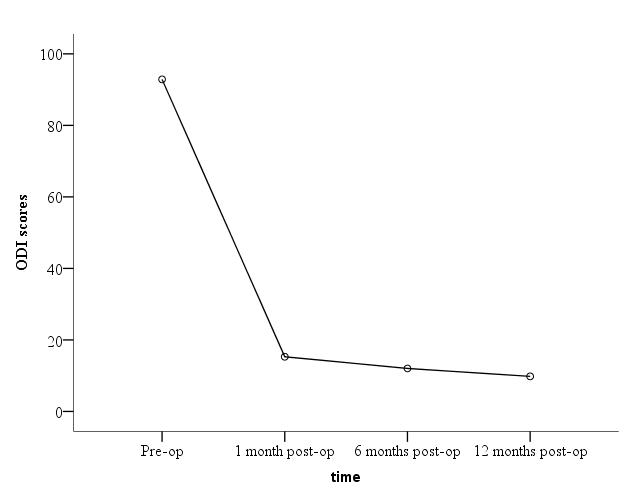


**Supplementary Fig 3**. Oswestry Disability Index (ODI) for functional improvement over time.
